# Developed a New Radiation Shielding Absorber Composed of Waste Marble, Polyester, PbCO_3_, and CdO to Reduce Waste Marble Considering Environmental Safety

**DOI:** 10.3390/ma15238371

**Published:** 2022-11-24

**Authors:** M. I. Sayyed, Mansour Almurayshid, Fahad I. Almasoud, Amjad R. Alyahyawi, Sabina Yasmin, Mohamed Elsafi

**Affiliations:** 1 Department of Physics, Faculty of Science, Isra University, Amman 11622, Jordan; 2 Department of Nuclear Medicine Research, Institute for Research and Medical Consultations (IRMC), Imam Abdulrahman bin Faisal University, P.O. Box 1982, Dammam 31441, Saudi Arabia; 3 Nuclear Science Research Institute, King Abdulaziz City for Science & Technology (KACST), P.O. Box 6086, Riyadh 11442, Saudi Arabia; 4 Department of Soil Sciences, College of Food and Agricultural Sciences, King Saud University, P.O. Box 2460, Riyadh 12372, Saudi Arabia; 5 Department of Diagnostic Radiology, College of Applied Medical Sciences, University of Ha’il, P.O. Box 2440, Ha’il 81451, Saudi Arabia; 6 Centre for Nuclear and Radiation Physics, Department of Physics, University of Surrey, Guildford GU2 7XH, UK; 7 Department of Physics, Chittagong University of Engineering and Technology, Chattogram 4349, Bangladesh; 8 Physics Department, Faculty of Science, Alexandria University, Alexandria 21511, Egypt

**Keywords:** marble, radiation shielding, radiation protection efficiency, half-value layer

## Abstract

The usage of radiation is mandatory for modern life; in the same manner, controlling the outflow of harmful radiation is vital and could be achieved via employing a shielding material to eliminate any potential nuclear and radiation accidents and incidents. Considering this point, this study aims to manufacture composite samples based on waste marble as novel radiation shields. The physical and radiation shielding ability of the prepared shields were determined and analyzed. For this purpose, a high-purity germanium (HPGe) detector was used to detect the incoming photons emitted from three point sources (Am-241, Cs-137, and Co-60). The radiation attenuation factors for the new marble-based composites were measured for some energies, ranging from 0.06 to 1.333 MeV. We examined the effect of increasing the PbCO_3_ and CdO contents on the physical properties and radiation attenuation factors of the newly developed radiation shielding absorber. We found that the density of the samples increases from 1.784 to 1.796 g/cm^3^ when the CdO changes from 0 to 12.5 wt%. The linear attenuation coefficient (LAC) for all marble compositions has the maximum value at 0.06 MeV, while the LAC decreases with increasing energy. The highest LAC was found for Marb-3, with a composition of waste marble (50 wt%), polyester (25 wt%), PbCO_3_ (17.5 wt%), and CdO (7.5 wt%). We studied the impact of the addition of CdO on the expense of PbCO_3_ and we found that the half value layer (HVL) decreases with increasing the CdO content. Hence, when there is no space problem, the newly developed radiation shielding absorber can be used to maintain the cost effectiveness and environmentally friendliness of products.

## 1. Introduction

To encounter the requirements to limit the negative impacts of radiation, especially for those working in the radiation zone, research into developing suitable radiation shielding materials is advancing every day. Since radiation is used widely in space programs, medical research, and other uses, shielding materials must be created in the necessary shapes to avoid exposure to neutron and gamma radiation because both radiations can occur simultaneously [1,2,3]. It is important to state that the harm caused by radiation to devices or persons in the radiation environment is strongly correlated with the amount of absorbed dose, time of exposure in the radiation zone, and the energy of radiation. Ionizing radiation is a type of high-energy photon that results from nuclear decay and has enough energy to ionize the electrons in atoms [4,5,6]. Non-ionizing radiation can cause a variety of health issues, just like ionizing radiation does, despite the fact that it is not as energetic. When exposed to the human body, neutrons, another type of ionizing radiation listed with gamma radiation, can likewise offer a risk that can have catastrophic, irreversible effects. Since the kind and shape of the shielding material are crucial to shield the emitted photons, the diversity in the uses of nuclear technology has necessitated improved and varied types of radiation shielding materials [7,8,9,10]. Therefore, it is crucial to have shielding materials that are flexible, strong, easy to shape, and affordable. These properties are provided by adding different elements and alloys to polymer materials. The sort of radiation that needs to be blocked determines how the material is prepared. Materials having low atomic number elements, such as polymers or marbles, must be employed if the material is to be used to attenuate neutron radiation. On the other hand, materials made of elements with large atomic numbers must be chosen if they are to be utilized to shield gamma radiation. The composite must be constructed with a blend of both categories in order to minimize both with the same material. There are numerous works in the literature for this aim [11,12,13]. Ozkalaycı et al. [14] studied polymers with PbCl_2_ for radiation shielding utilizations in the range of 59.5–1408 keV. Their findings demonstrated that the addition of PbCl_2_ enhanced the material’s radiation shielding effectiveness. Additionally, according to Ogul, polymer composites are practical materials that can assist the industrial sector in creating the needed radiation attenuator [15]. In previous research, Belgin [16] reinforced unsaturated polyester with high-density materials to explore the properties of gamma attenuation, and it was found that sensitive and reliable results could be obtained using experiments. Natural rubber/Sb_2_O_3_ composites were used by Yonphan and colleagues [17] to create flexible radiation shielding materials, and they observed that these composites have greater gamma attenuation properties than concrete, commercial windows, and red brick. Alzahrani et al. [18] investigated the nuclear shielding characteristics of alloys based on W, Fe, Ni, and Pb and reported on the gamma and neutron shielding qualities of the chosen alloys. They claimed that while there is no discernible difference between the chosen alloys for neutron attenuation, W-based alloy exhibits better gamma shielding. Furthermore, boron (B) was doped into a glass composite by Khong et al. [19], and they claimed that B improves the manufactured composite’s neutron shielding properties. In a similar manner, Kaewkhao and coworkers [20] examined the neutron shielding characteristics of boron-doped glass systems and emphasized the effectiveness of boron addition to glass samples as a neutron shield. In replacement of cement, the addition of waste marble into the concrete mixture amplified the durability and dimensional stability of mortar [21]. Marble waste mix with concrete showed greater potency comparing concrete mixed with cement [22]. Al-Buriahi et al. (2022) have shown that enhancement of the CdO content (molar concentration of 0–15%) in the newly settled xCdO—20PbO–10TeO_2_—(69-x) B_2_O_3_–1CuO glass system boosted up the shielding ability, reducing the effect of the photon, fast, and thermal neutron, which was higher comparing a few traditional and lately researched glass shields [23]. In the same year (2022), Saeed et al. studied the performance of heavy oil fly ash (HOFA) in epoxy for identifying mechanical, thermal, and radiation shielding properties. Their outcomes proved the likelihood of strengthening the epoxy resin’s thermal, mechanical, and radiation shielding properties, and that using the addition of the leftover heavy oil fly ash (HOFA) into the epoxy has a valued impact on construction, piping, and automobiles [24]. In 2017, Pande et al. found out that the incorporation of red mud into the polymer matrix markedly upgraded the shielding efficacy of the polymer matrix. There are two benefits to using red mud in the polymer matrix: one is an upright substitute as a radiation absorber, and another one is an achievable mode of waste disposal [25]. In 2020, Rammah et al. studied the impact of the heavy metal oxides (PbO + Bi_2_O_3_) on glass systems to reduce the effects of incident proton and alpha particles, γ-photons, and neutrons. The addition of (PbO + Bi_2_O_3_) to the glass systems increases the density of the glasses and enhances the shielding capacity of the glass system [26]. Every year, 180,000 ton waste marble powder is produced through construction globally. Acid rain, which contains H_2_SO_4_, in contact with waste marble powder (CaCO_3_) produce a carbon dioxide gas, which affects our ecosystems [27]. As a continuation of earlier efforts, the aim of this study is to develop a new radiation shielding absorber composed of waste marble, polyester, PbCO_3_, and CdO by utilizing the waste marble, considering cost effectiveness, where polyester is used, and flexibility, as well as PbCO_3_ and CdO as additives to enhance the radiation shielding ability. The physical and radiation shielding ability of this newly developed radiation shielding absorber are determined and analyzed.

## 2. Materials and Methods

### 2.1. Materials

#### 2.1.1. Liquid Polyester

The transparent polyester liquid belongs to the unsaturated polyester resin. It is a polyester used in the casting process and has a crystalline transparency during use. The liquid polyester is usually produced from a chemical reaction between ethylene glycol, an oil-based substance, and terephthalic acid. Polyester liquid is used in almost all industries, including in the production of industrial marble. The physical properties of used polyester have been reported in Table 1.

#### 2.1.2. Waste Marble

**Figure 1:**
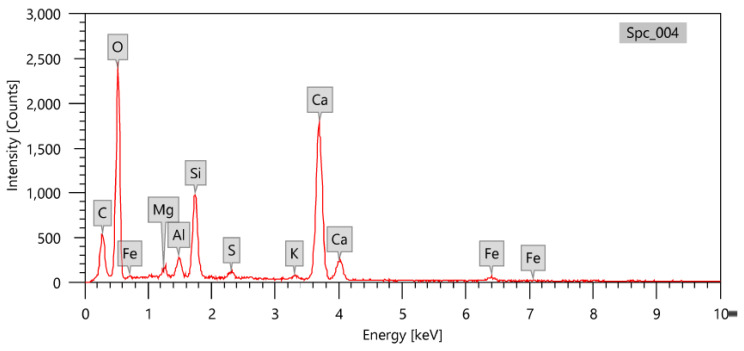
EDX analysis of the used waste marble.

The waste marble was produced from the marble factories through the operations of cutting, sawing, polishing, and leveling the marble surfaces. This waste marble was divided into large solid wastes of different sizes, the average size being 5 ± 2 mm; they were heavy viscous liquid wastes and dense emissions of dust with fine particles resulting from the volatilization of fine particles. Large solid waste marble of different sizes were used in this study and analyzed using EDX analysis to find out the average percentage of each element, as shown in Figure 1; their composition is shown in Table 2.

#### 2.1.3. Lead Carbonate (PbCO_3_)

Lead carbonate is considered one of the main components in the manufacturing of artificial marble, as it is dissolved with polyester because it has a high covering and adhesion ability; its density is 6.6 g/cm^3^ and it has an average size is 50 μm. However, one of its most important disadvantages is that it is toxic and therefore the main goal of this study is to replace or at least reduce its percentage in the mixture.

#### 2.1.4. Cadmium Oxide (CdO)

Cadmium oxide is considered one of the heavy oxides, with a density of 8.15 g/cm^3^ and an average size of 40 μm; in addition, it has a high absorption point (K-edge) at low energies and is a good absorber of neutrons, therefore being a good alternative to lead carbonate.

### 2.2. Sample Preparation

The polyester (Table 3) was mixed with lead carbonate according to the displayed proportions. It was stirred well, and then the marble waste (small crumbly pebbles) was added, the powdered cadmium oxide was added, the hardener was added at 5% of the polyester, stirred well, placed in cylindrical molds of 8 cm in diameter, and left for two days until it dried and gave a new type of composite.

### 2.3. Radiation Measurements

**Figure 2:**
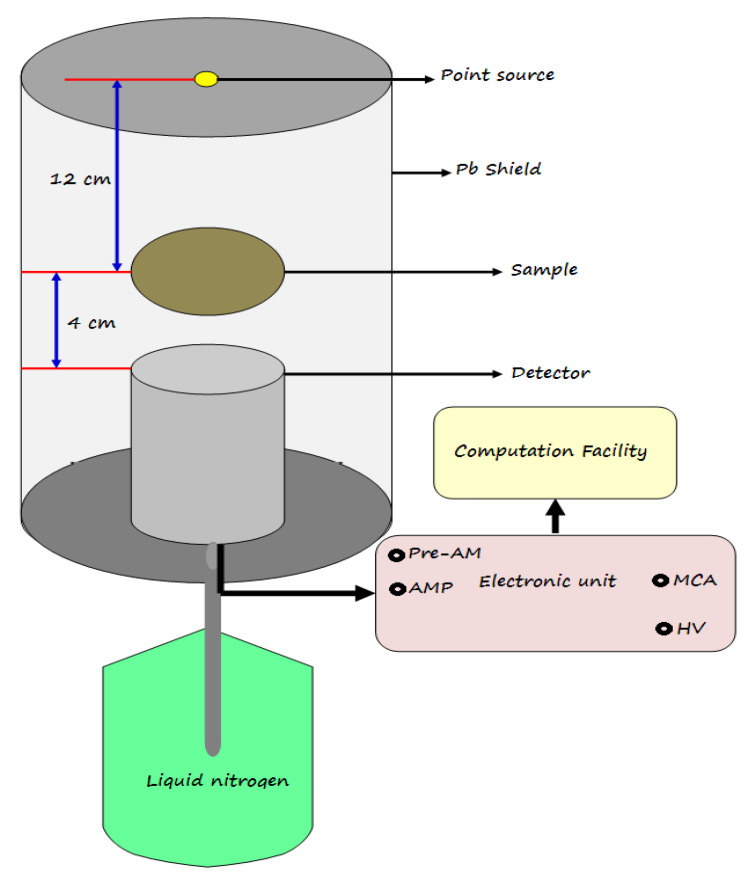
The geometry of the experimental work.

The attenuation properties of the present artificial marble samples were determined experimentally by using an HPGe detector with a resolution of 1.92 at 1.333 MeV and relative efficiency of 24%. Point sources were used covering the energy range, as Americium-241 gives a line of 0.060 MeV, Cesium-137 gives a line of 0.662 MeV, while Cobalt-60 gives two lines of energy of 1.173 and 1.333 MeV [28]. The absorbed sample was placed between the source and the detector at an appropriate distance that was calibrated, as shown in Figure 2. The measurement was made for a sufficient time in the presence (A) and absence $\left(A_{0}\right)$ of the sample for all sources with the same conditions to obtain different peaks related to incident energy photons, whose areas were calculated using the Genie 2000 program. From the calculated areas, the transmission factor (*TF*) can be experimentally calculated by Equation (1) [29].

(1) $$TF\;\left(\%\right)=\frac{I}{I_{0}}\times 100=\frac{A}{A_{0}}\times 100$$

The linear attenuation coefficient (*LAC*) represents the interaction potential of a photon inside the prepared marble sample through a certain pathlength (x) and it can be calculated by the following law [30]:

(2) $$LAC=\frac{1}{x}\;Ln\frac{A_{0}}{A}$$

The experimental values were compared by the Phy-X online software used for radiation attenuation parameters calculation [31]. The thicknesses needed to reduce the estimated area to its half and tenth values are called the half value layers (*HVL*) and are given by [32,33,34,35,36,37]:

(3) $$HVL=\frac{Ln\;2}{LAC}\;$$

## 3. Results and Discussion

There are many different forms of radiation and utilizations, and humanity benefits from these. Gamma radiation is the most penetrating form of radiation, making it the most challenging to minimize the number of gamma photons entering a medium by absorption or scattering. Although they theoretically cannot be totally stopped, they can be attenuated. Traditionally, gamma protection materials are a good solution in this situation, but because of their physical and chemical drawbacks, researchers have been looking for lighter, more affordable alternatives. This paper demonstrates the gamma shielding characteristics of new composites made at fixed amounts of waste marble (50 wt%) and polyester (25 wt%) with numerous amounts of PbCO_3_ ((25–12.5) wt%) and CdO ((0–12.5) wt%) dopant in order to suggest a new polyester composite for attenuating gamma radiation.

**Figure 3:**
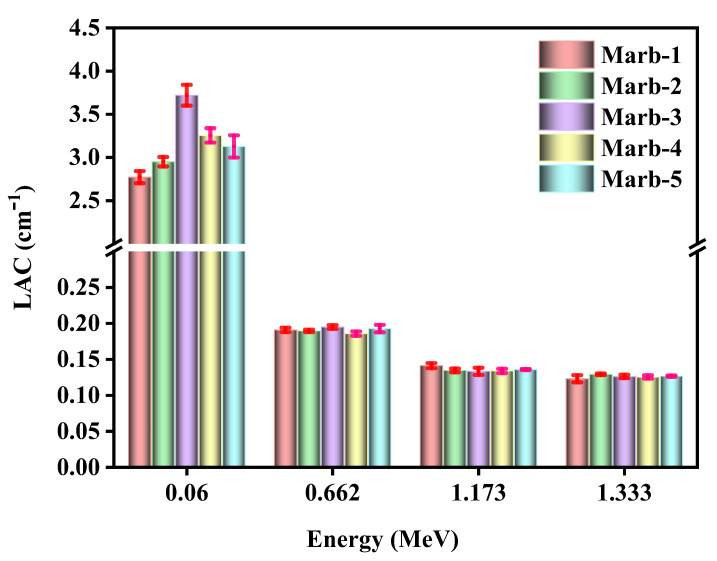
The demonstration of LAC of the prepared composites at energy 0.06 MeV, 0.662 MeV, 1.173 MeV, and 1.333 MeV.

At first, the value of the linear attenuation coefficients (*LAC*) of the prepared composites were measured using an HPGe detector at energy 0.06 MeV, 0.662 MeV, 1.173 MeV, and 1.333 MeV. The value of the linear attenuation coefficients with error bar (*LAC*) of the prepared composites against incident photon energy is represented in Figure 3 and Table 4. From this figure, it has been found that at energy 0.06 MeV, Marb-3 (polyester (25 wt%), waste marble (50 wt%), PbCO_3_(17.5 wt%), and CdO (7.5 wt%)) has characterized the highest value of LAC compared to the rest of the prepared composites; nevertheless, negligible variation was found at energy 0.662 MeV, 1.173 MeV, and 1.333 MeV.

**Figure 4:**
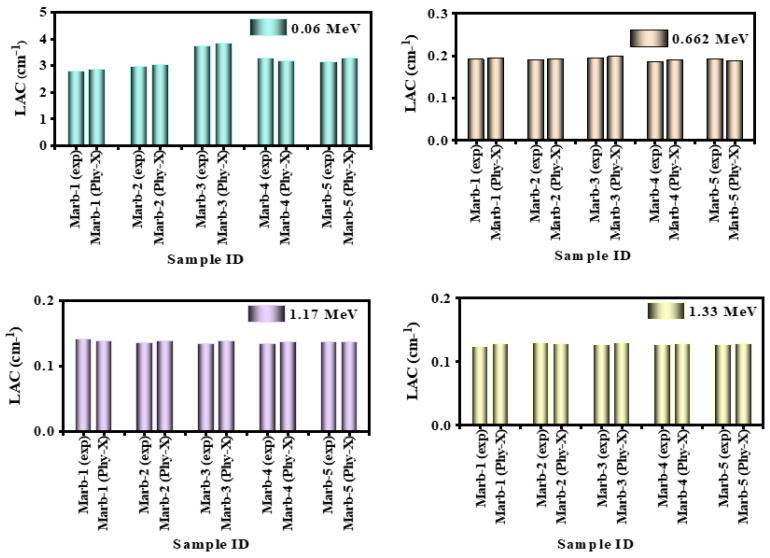
Demonstration of the deviation of the *LAC* values between the experimental and Phy-X software values.

For the purpose of validating our measured LAC values, the LAC for the corresponding composites was calculated theoretically using Phy-X, and then we compared the LAC produced from the two approaches. We calculated the deviation between the two approaches and using the deviation, we assessed the precision of our experimental design. The accuracy of our measured LAC values can be confirmed if the deviation is small (let us say less than 10%). This is a crucial step because, as we will see in the following paragraphs, various shielding factors can be inferred from the LAC. Figure 4 displays the LAC results obtained from both methods. The prepared composites reported the following deviation values: Marb-1(1.58–4.11) wt%, Marb-2 (0.97–1.97) wt%, Marb-3(1.35–3.77) wt%, Marb-4 (1.65–2.55) wt%, and Marb-5 (0.58–4.11) wt%; it is clear that the measured LAC values and the Phy-X results correspond rather well.

**Figure 5:**
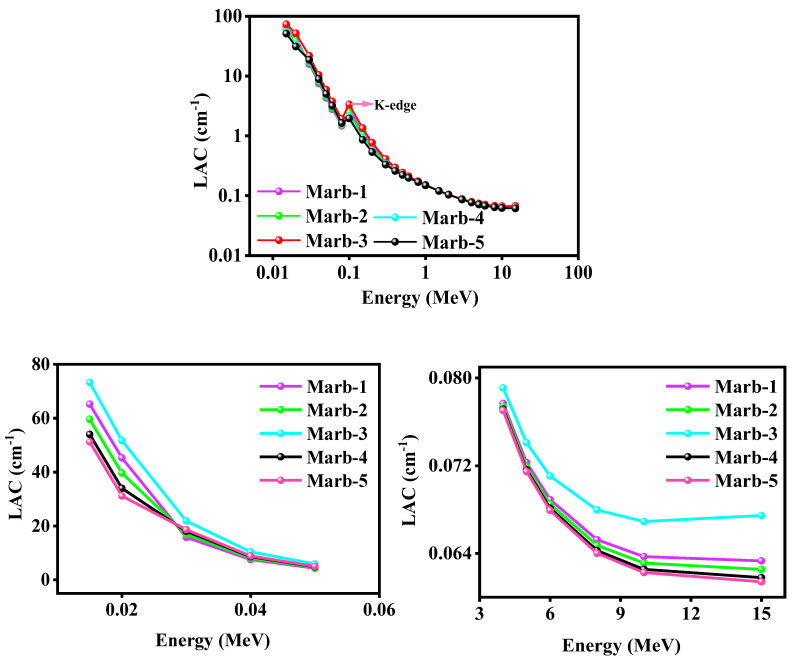
Demonstration of the LAC of the prepared composites.

The linear attenuation coefficients (*LAC*) of the prepared composites made up of polyester/waste marble/PbCO_3_/CdO at energies 0.015 MeV to 15 MeV were calculated by Phy-X software and the obtained values are presented in Figure 5. Herein, Figure 5 has demonstrated that the value of the linear attenuation coefficients (*LAC*) of the prepared composites declined with the rise in incident gamma radiation energy. Photoelectric absorption (PE) and Compton scattering (CS) can all be used to explain this tendency. It is because the PE mode is dominant at 0.06 MeV, while CS is dominant at the last two energies, 1.173 MeV and 1.333 MeV. The composition of Marb-3 provides better results despite having a similar density due to the k-edge effect of both Cd and Pb together. In the first compositions (Marb-1 and Marb-2) the concentration of Cd decreases, while in the latter compositions (Marb-4 and Marb-5) the concentration of Pb decreases.

To understand the shielding ability of any absorber, HVL is one of the most valid parameters. The value of HVL was measured for the prepared composites (polyester/waste marble/PbCO_3_/CdO) using the Phy-X software in the energy range from 0.015 MeV to 15 MeV. It is well established that the K-edge of lead is 88 keV, whereas Figure 5 shows that the value of the k-edge of the studied lead sample was ~100 KeV. It indicates that the studied sample showed better shielding ability at energy100 KeV.

**Figure 6:**
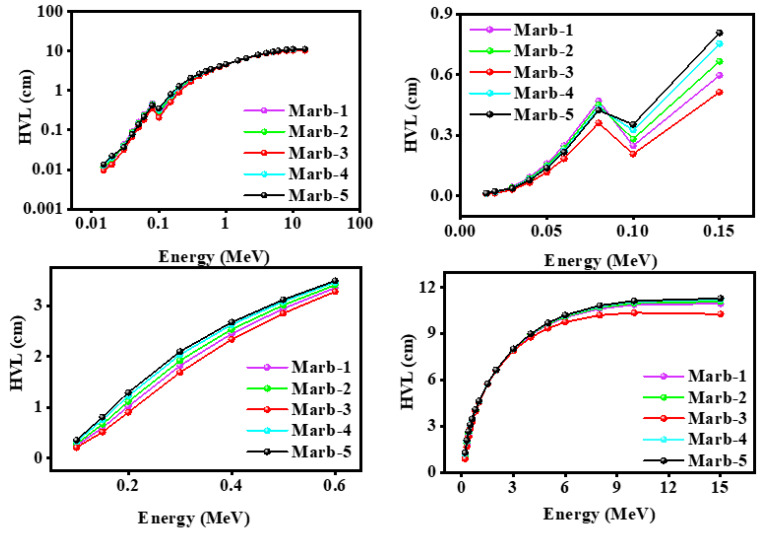
Illustration of the HVL of the prepared composites (polyester/waste marble/PbCO_3_/CdO) against incident photon energy.

Figure 6 provides the HVL of the prepared composites (polyester/waste marble/PbCO_3_/CdO) against incident photon energy. The results revealed that from energies of 0.015–0.6 MeV and 3–15 MeV, Marb-3 gave the lowest value of HVL, whereas from 0.8–3 MeV negligible variation was found. Therefore, it can be stated that the prepared composite Marb-3 had the best shielding ability of the prepared samples.

**Figure 7:**
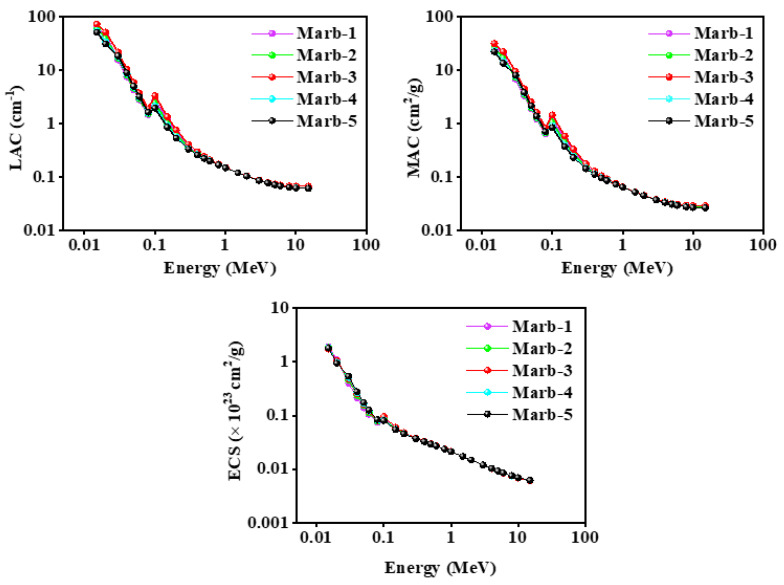
The theoretical values of LAC, MAC, and ECS of the prepared composites are within the energy limit of 0.015 MeV to 15 MeV.

Figure 7 shows the movement of the value of linear attenuation coefficient (LAC), mass attenuation coefficient (MAC), and electronic cross-section values of the photons (ECS) of the prepared composites against incident photon energy from 0.015 MeV to 15 MeV. The obtained values revealed that the value of the linear attenuation coefficient (LAC), mass attenuation coefficient (MAC), and electronic cross-section values of the photons (ECS) of the prepared composites has declined with the increase in incident photon energy. Moreover, they maintained a very similar inclination.

**Figure 8:**
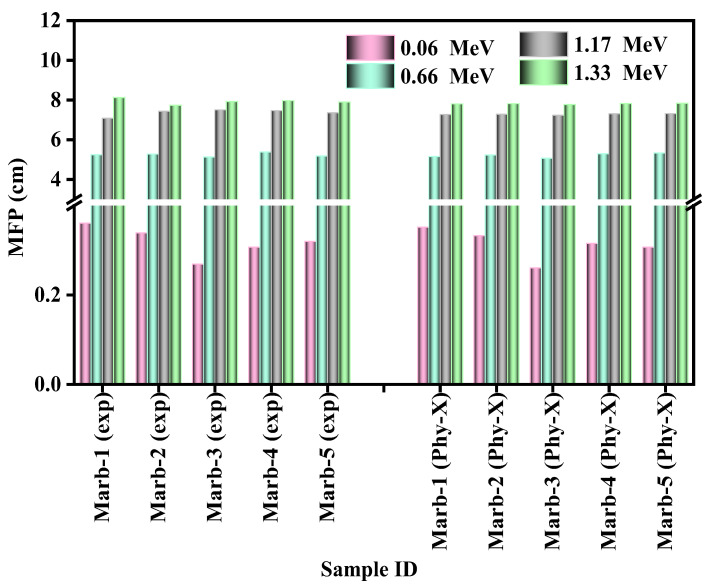
The comparison between the experimental and theoretical values of the MFP of the prepared composites.

MFP is a pointer to specify the shielding efficacy of any absorber, which is the traveled distance between the interactions of two possible photons for that absorber. The reciprocal value of the linear attenuation coefficient is the MFP. Through the lowest MFP values, an absorber represents its shielding ability. Contrariwise, for higher incident photon energy, to reduce the hazards of radiation, a thicker shielding absorber is required. A comparison between the experimental and theoretical values of the MFP of the prepared composites is displayed in Figure 8. This figure epitomizes the outstanding corroboration between the theoretically and experimentally evaluated MFP values of the prepared composites. This figure also undoubtedly shows that the value of MFP is amplified according to the enhancement in the incident photon energy from energy 0.06 MeV to 1.33 MeV. Among the five prepared composites, Marb-3 was revealed the have the lowest value of MFP. It is well established that a good shielding absorber represents the lowest value of MFP; hence, it can be said that Marb-3 has the greatest shielding ability compared to the rest of the prepared composites.

**Figure 9:**
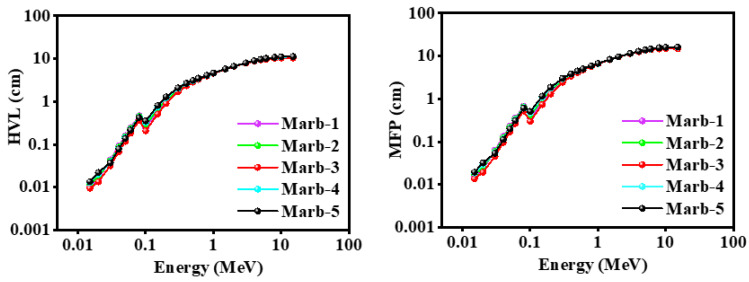
The theoretical value of the HVL and MFP of the prepared composites within the energy limit of 0.015 MeV to 15 MeV.

The theoretical value of HVL and MFP of the prepared composites within the energy limit of 0.015 MeV to 15 MeV is presented in Figure 9. These histograms revealed that the value of HVL and MFP of the prepared composites increased with the increase in incident photon energy; they also follow an approximately similar trend.

**Figure 10:**
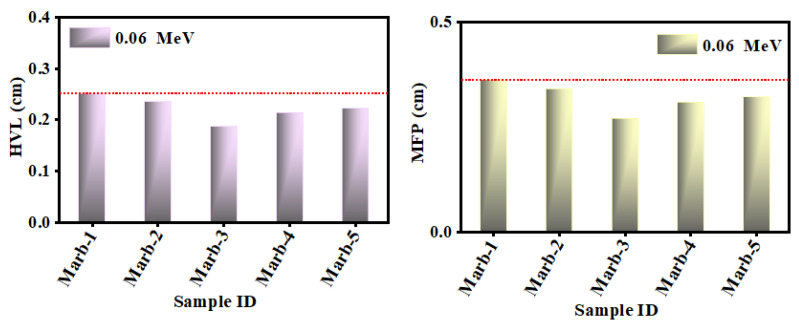
The value of the HVL and MFP of the prepared composites at energy 0.06 MeV.

For energy 0.06 MeV, the value of HVL and MFP of the prepared composites are represented in Figure 10. Marb-3 shows the lowest value of HVL and MFP. Hence, it can be mentioned that Marb-3 has the greatest shielding ability over all of the prepared composites. It is well known that Pb is a toxic element; yet, no other element has a greater shielding ability than Pb. That is why when any innovative shielding composites are being prepared, the Pb-elements have to be used to get better shielding ability. In this experimental finding, a noteworthy outcome was seen, where Marb-3 (17.5 wt% PbCO_3_ and 7.5 wt% CdO) has shown a better shielding ability than Marb-1 (25 wt% PbCO_3_). It is undoubted that Marb-3 has a lower amount of toxic Pb elements than Marb-1; hence, using Marb-3 as shielding material has maintained the minor risk but with a greater shielding efficiency.

**Figure 11:**
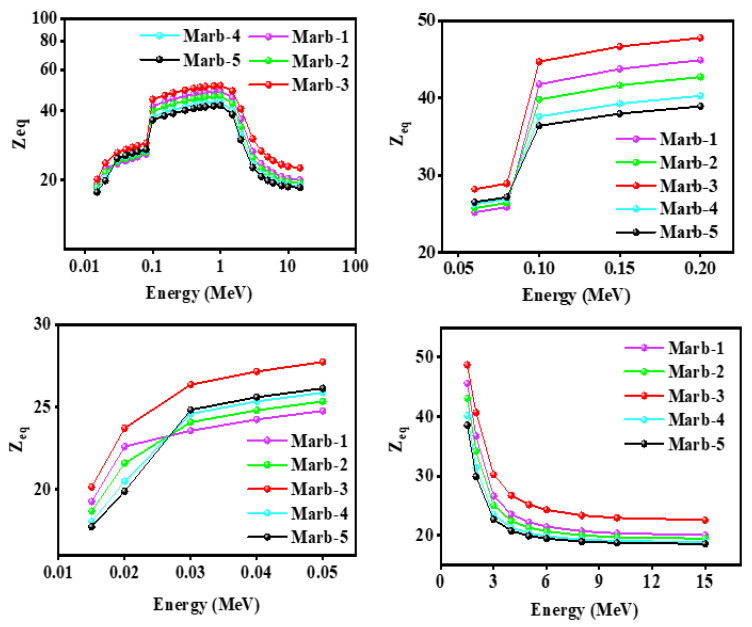
Illustration of the equivalent atomic number (Z_eq_) of the prepared composites (waste marble/polyester/PbCO_3_/CdO) against incident photon energy.

Equivalent atomic numbers (Z_eq_) of the innovative polyester samples (polyester/waste marble/PbCO_3_/CdO) are displayed in Figure 11. Within the energy limit of 0.015 MeV to 15 MeV, the prepared composite Marb-3 has shown the highest value of equivalent atomic numbers (Z_eq_). As an example, at energy 0.02 MeV, the highest value of equivalent atomic numbers was found for Marb-3 (47.7) whereas the lowest value for Marb-5(38.9). Hence, it is a plain explanation that, among the prepared samples, Marb-3 has the best shielding ability.

## 4. Conclusions

In order to get a better shielding ability with less toxicity, we developed a pioneer radiation shielding absorber composed of waste marble (50 wt%), polyester (25 wt%), PbCO_3_ ((25–12.5) wt%), and CdO ((0–12.5) wt%) to reduce waste marble, also considering environmental safety. According to the obtained lowest value of HVL and MFP of the prepared composites, sample Marb-3 had the best shielding ability. Moreover, in the energy limit of 0.015 MeV to 15 MeV, the prepared composite Marb-3 displayed the highest value of equivalent atomic numbers (Zeq). A remarkable upshot was found in that Marb-3 (17.5 wt% PbCO_3_ and 7.5 wt% CdO) had a better shielding ability than Marb-1 (25 wt% of PbCO_3_). It is certain that Marb-3 has a lower quantity of toxic Pb elements than Marb-1. Hence, it can be said that the utilization of Marb-3 will be safer than the rest of the prepared samples, and it has a better shielding performance as well. The main aim of this study was to show how one can utilize the huge amount waste marble being produced all over the world each year. That is why the greatest amount of waste marble of (50 wt%) was used to produce the newly developed radiation shielding absorber. Hence, when there is no space problem, the newly developed radiation shielding absorber can be used to maintain the cost effectiveness and environmentally friendliness of products.

## Figures and Tables

**Table 1:** The physical properties of the used polyester resin.

Density	1.25 g/cm^3^
Yield modulus	2–4 GPa
Compressive Strength	140 MPa
Tensile Strength	55 MPa
Tensile Elongation at Break	2%

**Table 2:** The composition (%) of the waste marble.

Name of Oxide	Composition (%)
CaO	35.24
Fe_2_O_3_	0.23
MgO	5.27
Al_2_O_3_	3.55
SiO_2_	11.5
SO_3_	1.78
K_2_O	1.21
LOI	41.22

**Table 3:** Chemical composition of the produced new radiation shielding absorber.

Codes	Compositions (wt%)				Density (g/cm^3^)
	Waste Marble	Polyester	PbCO_3_	CdO	
Marb-1	50	25	25	0	2.291
Marb-2	50	25	20	5	2.298
Marb-3	50	25	17.5	7.5	2.302
Marb-4	50	25	15	10	2.306
Marb-5	50	25	12.5	12.5	2.311

**Table 4:** The measured and theoretical LAC of the prepared samples.

Code	Energy (MeV)	*LAC* (cm^−1^)		Unc (Exp)	R.D (%)
		Phy.X	Exp		
Marb-1	0.060	2.8418	2.7720	0.0011	2.52
	0.662	0.1939	0.1909	0.0009	1.58
	1.173	0.1377	0.1412	0.0031	−2.54
	1.333	0.1282	0.1231	0.0018	4.11
Marb-2	0.060	3.0061	2.9509	0.0008	1.87
	0.662	0.1914	0.1895	0.0024	0.97
	1.173	0.1372	0.1346	0.0014	1.97
	1.333	0.1279	0.1292	0.0007	−0.99
Marb-3	0.060	3.8407	3.7202	0.0009	3.24
	0.662	0.1976	0.1949	0.0025	1.35
	1.173	0.1384	0.1333	0.0017	3.77
	1.333	0.1287	0.1262	0.0013	1.98
Marb-4	0.060	3.1727	3.2557	0.0010	−2.55
	0.662	0.1889	0.1858	0.0016	1.65
	1.173	0.1369	0.1339	0.0008	2.22
	1.333	0.1277	0.1255	0.0008	1.75
Marb-5	0.060	3.2564	3.1279	0.0014	4.11
	0.662	0.1876	0.1928	0.0019	−2.65
	1.173	0.1367	0.1359	0.0020	0.58
	1.333	0.1276	0.1266	0.0030	0.79

## Data Availability

All relevant data are within this paper.
